# Flexibility-powered Pickering emulsion enhances mucus permeability to alleviate ulcerative colitis

**DOI:** 10.1186/s12951-025-03829-6

**Published:** 2025-12-23

**Authors:** Jiali Lv, Chen Cheng, Xinran Liu, Jinhua Song, Daxiang Li, Yijun Wang, Yiqun Du

**Affiliations:** 1 State Key Laboratory of Tea Plant Germplasm Innovation and Resource Utilization, School of Food and Nutrition, Anhui Agricultural University, Hefei, 230036 China; 2 Institute of Clinical Immunology, the First Affiliated Hospital of Anhui Medical University, Anhui Medical University, Hefei, 230032 China

**Keywords:** Flexibility deformation, Pickering emulsion, Ulcerative colitis, Oral delivery, Chitosan, EGCG

## Abstract

The physicochemical properties of oral drug delivery carriers significantly influence their interactions with the intestinal barrier, which, in turn, affect their in vivo fate and therapeutic efficacy. As a delivery system with a hierarchical structure and high stability, Pickering emulsions have demonstrated significant advantages in enhancing the bioavailability of encapsulated bioactive compounds. However, the specific role of their inherent flexibility in modulating interactions with the intestinal barrier and influencing delivery efficiency remains poorly understood. In this study, a flexible Pickering emulsion (FPPE) stabilized by chitosan nanoparticles was developed for encapsulating epigallocatechin gallate, aiming to investigate the role and underlying mechanism of its flexible structure in mucus penetration, cellular uptake, and biodistribution in the intestinal tract. Upon reaching the intestinal tissue, FPPE exhibited deformable adaptation, facilitating efficient mucus permeation, prolonged gastrointestinal retention, and enhanced drug absorption. Moreover, FPPE enhanced macrophage uptake and mitochondrial membrane potential, reduced reactive oxygen species scavenging, and triggered macrophage reprogramming. Compared to rigid chitosan microspheres, FPPE more effectively alleviated intestinal inflammation and promoted intestinal barrier repair in a dextran sulfate sodium-induced acute colitis mouse model. This study reveals how Pickering emulsion flexibility facilitates intestinal mucus delivery, guiding their future design and application.

**Graphical Abstract:**

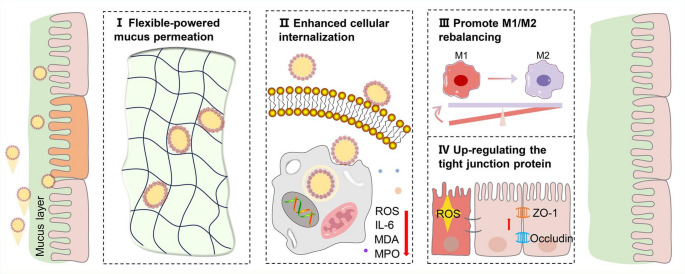

**Supplementary Information:**

The online version contains supplementary material available at 10.1186/s12951-025-03829-6.

## Introduction

Ulcerative colitis (UC) is a nonspecific inflammatory disease characterized by colonic and mucosal inflammation [1–3]. Its recurrent nature makes effective treatment challenging and highlights the need for safe, efficient, and cost-effective treatment strategies [4, 5]. Foodborne functional ingredients can effectively treat UC by minimizing the side effects and recurrence rates of Western medicine, making their development a promising strategy for the prevention and treatment of UC [6–8]. However, poor gastrointestinal stability and the lack of targeting of foodborne functional ingredients hinder their effectiveness in treating UC [9, 10]. Thus, a food delivery system is required to incorporate bioactive molecules into nano- and micro-carriers to enhance stability and facilitate targeted delivery, thereby improving bioavailability and nutritional absorption [11–13].

Research on food delivery systems has shown that the structural characteristics of delivery carriers, including particle size, surface charge, specific surface area, and particle shape, can substantially affect their interactions with the intestinal tissues and barriers [14–17]. Specifically, the diverse properties of these carriers can result in varying localization and distribution patterns within the intestinal mucosa and mucus, influencing their barrier crossing, membrane binding, and intracellular uptake, which affects targeted delivery efficacy [18]. Recently, Liu et al. reported that mesoporous silica nanorods exhibited a higher diffusion rate in mucus than conventional nanorods, thereby enhancing drug retention and absorption at the intestinal surface [19]. Guo et al. demonstrated that the absorption and uptake of particles measuring 100 nm in size in the small intestine were 1.38 times greater than those of particles measuring 500 nm [20]. In addition to research emphasizing the size, shape, and surface characteristics of delivery carriers in relation to their in vivo delivery performance, the elasticity of these carriers represents another significant physicochemical parameter that exhibits a unique contact elastic deformation effect [21–23]. Given that the flexible contact properties of a delivery vehicle can be modulated, flexible contact deformation under biological forces may influence the cellular uptake in vivo and interactions with biological targets. Notably, Takechi-Haray et al. observed that liposomes with greater bending rigidity are internalized more rapidly by HeLa cells than liposomes with lower rigidity [24]. Despite considerable effort to elucidate the influence of particle elasticity on blood circulation and cellular uptake mechanisms, the role of particle flexibility in intestinal mucus permeation remains unclear.

Pickering emulsions represent innovative formulations stabilized by nanoparticles that do not incorporate surfactants [25, 26]. Upon the formation of the Pickering emulsion, the interfacial dissociation energy of the nanoparticles at the emulsion surface significantly exceeds the thermal energy associated with the intrinsic movement of the nanoparticles [27]. This allows the nanoparticles to remain anchored at the oil–water interface of the emulsion. Consequently, in contrast to traditional surfactant-stabilized emulsions, Pickering emulsions exhibit superior stability, fewer side effects, and improved safety [28–30]. Importantly, our previous study demonstrated that Pickering emulsions exhibit distinctive flexibility and deformability, making them an exceptional delivery system [31].

**Fig. 1:**
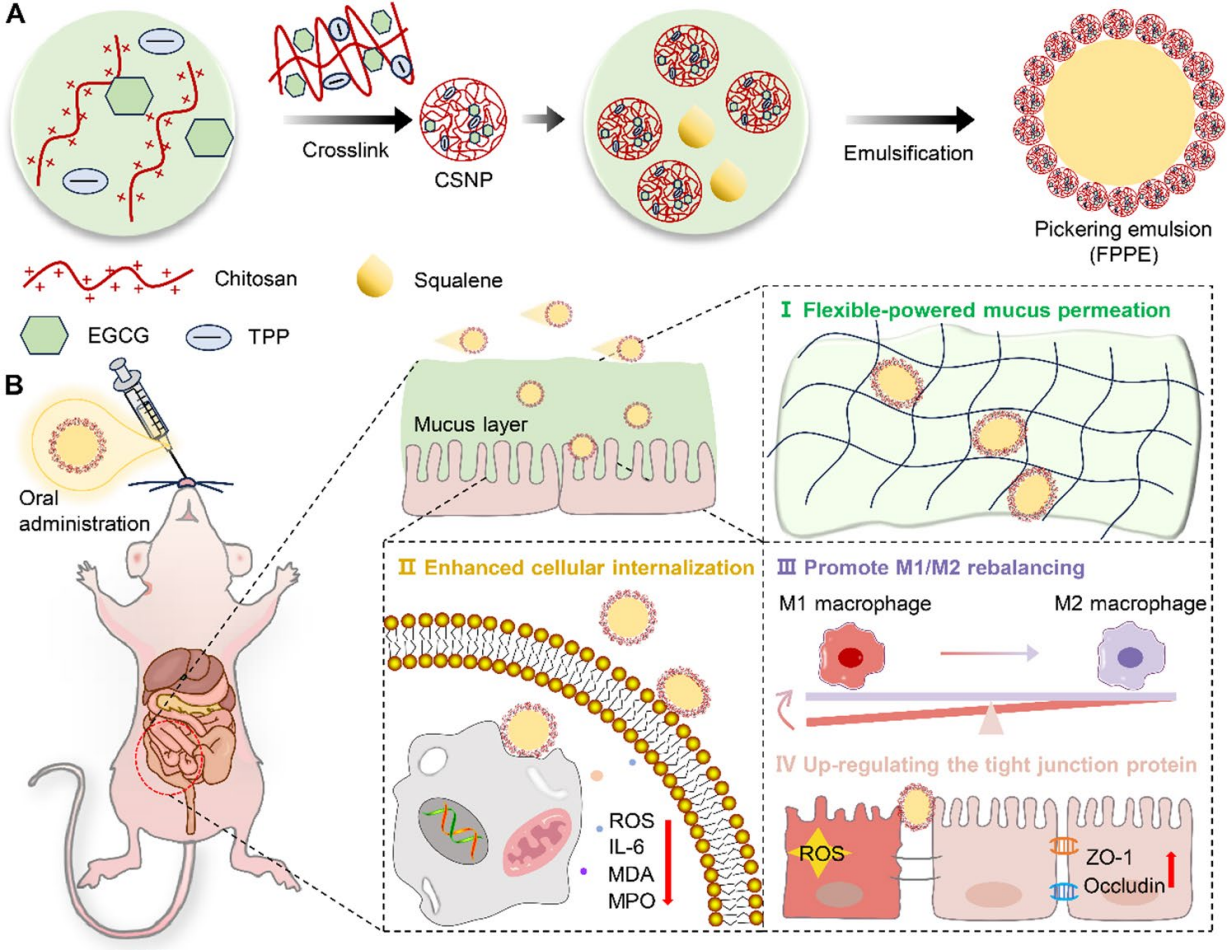
Mechanism diagram of the penetration and uptake of flexible Pickering emulsion in intestinal mucus. (**A**) Schematic illustration of the preparation process of FPPE. (**B**) FPPE was orally administered via gavage, and soft contact deformation structure enabled efficient penetration through the intestinal mucus barrier, leading to its accumulation in the inflamed colon. The efficient osmotic adsorption capacity of FPPE alleviated intestinal inflammation and facilitated the restoration of the integrity of the damaged intestinal barrier

Based on the above considerations, we developed a flexibility-powered Pickering emulsion (FPPE) to efficiently deliver epigallocatechin gallate (EGCG) and investigated the role of its elastic properties in intestinal mucus penetration (Fig. 1). EGCG, a natural phenolic compound present in various foods and beverages (particularly tea), is known for its extensive range of biological activities, including antioxidant, anti-inflammatory, antiobesity, and anticancer effects [32–35]. To ensure a strong biosafety profile, biodegradable chitosan nanoparticles and the natural lipid squalene were employed as stabilizers and oil cores, respectively. EGCG was encapsulated in chitosan nanoparticles to enhance its intestinal stability and provide controlled release properties. Mucus permeation was enhanced by exploiting the deformability of the Pickering emulsions. Our study aimed to elucidate the differences between flexible and rigid delivery carriers in terms of their permeation through the intestinal mucus. This study provides guidance for the rational design of targeted drug delivery systems for intestinal applications.

## Materials and methods

### Materials

Chitosan (≥ 95% deacetylation), sodium tripolyphosphate, epigallocatechin gallate (EGCG), squalene, coumarin-6, and amino-Cyanine5 (Cy5) were purchased from Aladdin (China). DIO and DAPI were purchased from Beyotime (Shanghai, China), while Hoechst 33342 and iFluor647-Wheat Germ Agglutinin (WGA) were sourced from Solarbio (Beijing, China). Cell lines RAW264.7 (CL-0696), Caco-2 (CL-0017), and HT-29 (CL-0012) were provided by Meilun Bio (Dalian, China). High-glucose Dulbecco’s modified Eagle’s medium (DMEM), fetal bovine serum (FBS), penicillin-streptomycin, and MEM non-essential amino acids solution were purchased from Gibco (USA). Phosphate-buffered saline (PBS) was obtained from Sangon Biotech (China). Dextran sulfate sodium (DSS, MW = 5000) was purchased from Sparkjade (China). C57BL/6J mice were obtained from GemPharmatech Co., Ltd (Jiangsu, China). Histological staining kits, including H&E staining, AB-PAS, MDA, and MPO activity kits, were obtained from Solarbio (Beijing, China). IL-6 Elisa kit was purchased from Yuanju Bio (China). All reagents used in this study were of analytical grade.

## Ethics statement

C57BL/6J male mice (6 weeks of age) used in this research were housed under specific pathogen-free conditions. Autoclaved chow and water ad libitum were used to feed all animals. All animal experimental protocols were reviewed and approved by the Institutional Animal Care and Use Committee of Anhui Agricultural University.

## Preparation and characterization of FPPE

Chitosan was dissolved in a 1.0% (v/v) acetic acid solution to prepare a chitosan acetate solution at a concentration of 1.5 mg/mL, with the pH subsequently adjusted to 5.0. EGCG was added to the chitosan solution in a 10:1 mass ratio of chitosan to EGCG and stirred in the dark for 30 min. Tripolyphosphate (TPP) was dissolved in ultrapure water at a concentration of 0.8 mg/mL. Chitosan nanoparticles (CSNP) were synthesized using an ionic crosslinking method by gradually adding TPP to the chitosan acetate solution at a chitosan to TPP mass ratio of 3:1 under continuous stirring. The resulting CSNP were then concentrated to 5 mg/mL via ultrafiltration, mixed with squalene, followed by ultrasonic emulsification to produce the flexibility-powered Pickering emulsion (FPPE). For the preparation of chitosan microparticles (CSMP), the concentrations of both chitosan and TPP were increased, and the rate of TPP droplet addition was modified accordingly.

The morphology of CSNP and CSMP was characterized using a Hitachi field emission scanning electron microscope (SU8600). Microstructural observations were conducted at accelerating voltages of 15 kV and 20 kV, focusing on representative regions. Particle size, zeta potential, and polydispersity index (PDI) of all particles were determined using a Zetasizer Nano Z (Malvern Instruments, Malvern, UK) at 25 °C with a fixed scattering angle of 90°. Additionally, the FPPE formulation was stored at 4 °C and 25 °C for 3, 6, 9, 12, and 15 days, during which the particle size and PDI were re-evaluated using the Zetasizer Nano Z.

The mechanical properties of FPPE and CSMP particles were evaluated using atomic force microscopy (AFM) (FastScan, Bruker, Germany). The obtained force curves were analyzed with NanoScope analysis software.

The encapsulation efficiency (EE) of EGCG in the nanoparticles was determined using high-performance liquid chromatography (HPLC). Briefly, the EGCG-loaded nanoparticle solution was transferred into a 10 kDa ultrafiltration tube and centrifuged at 3000 × g for 20 min. The filtrate was collected, and the EGCG content in the filtrate was measured by HPLC. The EE was calculated using the following formula:

$$\:EE\left(\%\right)=\frac{total\:amount\:of\:EGCG\:added-free\:EGCG}{total\:amount\:of\:EGCG\:added}\times\:100\%$$

## Cell lines and cell culture

The cell cultures were performed to support subsequent experimental procedures. RAW264.7 and HT-29 cells were cultured in DMEM supplemented with 10% fetal bovine serum (FBS) and 1% penicillin-streptomycin. Caco-2 cells were maintained in a medium containing 10% FBS, 1% non-essential amino acids, and 1% penicillin-streptomycin. All cell lines were kept in a humidified incubator at 37 °C with 5% CO_2_.

## Construction of a cell co-culture model and evaluation of mucus penetration ability

To establish a cell model with a mucus layer, Caco-2 and HT-29 cells were co-cultured in a 7:3 ratio at a density of 1 × 10^5^ cells·mL^− 1^ on confocal culture dishes for 14 days [36, 37]. After this incubation period, the spent medium was removed, and the co-culture was treated with Coumarin-6-labeled CSNP, CSMP and FPPE particles at 37 °C for 2 h. Following the incubation, the cells were washed three times with cold PBS to cease cellular uptake. The nuclei were stained with Hoechst 33,342, while the mucus layer was stained with Wheat Germ Agglutinin (WGA)-Alexa Fluor™ 647 conjugate. The samples were then analyzed using a confocal laser scanning microscope (CLSM) to evaluate the penetration of particles through the mucus layer.

### Evaluation of mucus penetration ability in the colon

To investigate the penetration of FPPE in the colon, C57BL6/J mice were sacrificed, and the colon was excised and rinsed with PBS to eliminate any contents. The colon was cut into 2 cm loops, and both ends were ligated using sterile sutures. Subsequently, 10 µL of WGA-Alexa Fluor™ 647 conjugates were added to the colon segments, which were then incubated in PBS at 37 °C for 30 min to stain the mucus layer. Following this, 200 µL of Coumarin-6 fluorescent dye-labeled FPPE was introduced and incubated for 1 h. After incubation, remove the sterile threads at both ends, drain the solution and wash it clean with PBS. The opened colon sections were then examined using CLSM to capture images of FPPE penetration within the mouse colon [38, 39].

### Cellular uptake of FPPE *in vitro*

RAW264.7 cells were seeded at a density of 1 × 10^6^ cells/mL in confocal culture dishes and cultured to the appropriate confluence. Cy5 fluorescent dye-labeled FPPE particles were then added to the dishes containing the RAW264.7 cells. The cells were co-incubated with FPPE for 2 h at 37 °C with 5% CO_2_. Following incubation, the culture medium was discarded, and the cells were washed with cold PBS to remove any non-phagocytosed particles. Cell membranes and nuclei were subsequently stained using DIO dye and Hoechst 33342, respectively. Confocal laser scanning microscopy was employed to capture images of RAW264.7 cells internalizing FPPE [40].

Moreover, RAW264.7 cells were cultured in 24-well plates at a density of 1 × 10^6^ cells per well. Cy5-labeled FPPE were added to the wells and incubated with RAW264.7 cells at 37 °C for time points of 10 min, 30 min, 1 h, and 2 h. After incubation, the cells were washed with cold PBS to remove uninternalized particles. Cells from each time point were collected and resuspended in flow cytometry buffer for analysis. Flow cytometry was performed using the APC channel on a Cyto-FLEX, with untreated cells serving as negative controls. Additionally, CLSM image analysis of FPPE uptake by Caco-2 cells was performed.

## Reactive oxygen species (ROS) and mitochondrial membrane potential assay

RAW 264.7 cells were also seeded in 24-well plates (glass bottom) at a density of 1 × 10^6^ cells per well. To induce ROS, cells were treated with LPS (200 µg/mL) and subsequently incubated with FPPE [41, 42]. Finally, cells were harvested, blocked, and labeled with DCFH-DA (10 µg/mL) staining solution for 30 min. In addition, the JC-1 staining solution was used for the labeling and detection of mitochondrial membrane potential. Images were captured using CLSM to visualize ROS generation and mitochondrial membrane changes [43].

## Macrophage M1/M2 polarization

For in vitro M1/M2 polarization, RAW 264.7 cells were seeded in 24-well plates at a density of 1 × 10^6^ cells per well. To generate M1 macrophages, cells were treated with LPS (200 µg/mL) followed by incubation with FPPE. After the treatment, cells were harvested, blocked, and stained with antibodies specific to the surface markers CD86 (M1) and CD206 (M2) for flow cytometric analysis to quantify the M1/M2 ratio [44].

### *In vivo* distribution of FPPE

Cy5-labeled FPPE particles were administered orally to C57BL/6J mice. Following administration, the entire gastrointestinal tract (from stomach to colon) was excised at 6, 12, 24 h post-administration. The fluorescent distribution of FPPE within the gastrointestinal tract was analyzed using an in vivo imaging system [45]. Mice were randomly divided into four groups, with each group consisting of three mice. A predetermined dosage of FPPE was administered to each group, and the gastrointestinal tracts were extracted at the specified time points for fluorescent distribution analysis. Prior to the experiment, C57BL/6J mice were fasted for 12 h but had free access to water.

### Visualization of intracellular uptake of FPPE *in vivo*

200 µL Coumarin 6-labeled FPPE particles were orally administered to C57BL/6J mice. After 12 h, the mice were sacrificed. The colon tissue was then extracted, rinsed with PBS, and fixed in 4% paraformaldehyde for 12 h. Subsequently, the tissue was embedded in a mixture of optimal cutting temperature compound (OCT) for frozen sectioning. Colon Sect. (10 μm) were stained with DAPI to label the nuclei, and the sections were examined using a CLSM to capture images of colon cells that had internalized FPPE [46].

### Colonic macrophage uptake of FPPE *in vivo*

Cy5-labeled FPPE particles were orally administered to C57BL/6J mice. Twelve hours post-administration, the mice were sacrificed, and colon tissues were harvested. The tissues were washed with PBS, and intestinal macrophages were isolated from the colon tissue. These macrophages were then stained with anti-mouse F4/80 antibody and resuspended in flow cytometry buffer for quantitative analysis of FPPE internalization. The internalization was assessed using a flow cytometer.

### Induction of experimental ulcerative colitis in mice

C57BL/6J mice, aged 6 weeks and of similar weight, were randomly divided into six groups: Healthy control group (Control), colitis control group (DSS), EGCG group (50 mg/kg/day) (DSS + EGCG), CSNP group (DSS + CSNP), CSMP group (DSS + CSMP), and FPPE group (DSS + FPPE). All animal were kept in a 12 h dark/light cycle at 25 °C. All animal experiments were conducted in strict compliance to the guidelines of the Institutional Animal Care and Use Committee of Anhui Agricultural University. Ulcerative colitis model was induced by feeding mice with aqueous DSS solution (3%) for 7 days, while the healthy control group received water [47].

Following the successful induction of colitis, all groups were switched to normal drinking water instead of DSS water, and the corresponding treatments were administered daily for an additional 7 days. During this period, the fecal consistency, fecal blood, and weight loss of mice were recorded daily to evaluate the disease activity index (DAI). We recorded the average of the total scores of the three results as the DAI value. The DAI value was calculated as the average of the total scores derived from these three parameters.

### Evaluation of body weight, colon and organs

After the 7-day treatment, all mice were euthanized, and their body weights were recorded prior to dissection. The entire colon was carefully excised, and its length from the ileum to the anal verge was measured as a key indicator of ulcerative colitis severity, with shorter lengths typically reflecting more severe inflammation and tissue damage. Additionally, major organs, including the heart, liver, spleen, and lungs, were carefully isolated, and their weights were recorded to evaluate potential systemic inflammation and organ-specific effects.

### Histologic evaluation of the colon and organs

For histopathological analysis, a small segment of colon (approximately 1 cm) was collected from each group of mice and fixed in 4% paraformaldehyde for 24 h. The fixed tissue was embedded in paraffin blocks and sectioned at a thickness of 5 μm using a rotary microtome (Leica RM2255). Hematoxylin-eosin (H&E) staining was performed to evaluate the extent of tissue damage, while Periodic Acid-Schiff (PAS) staining assessed the integrity of the colonic mucosa in colitis-affected mice [48]. Additionally, Alcian blue (AB) staining was conducted to analyze intestinal mucosal polysaccharide content. All stained paraffin sections were imaged using an inverted microscope to observe histological changes in the colons across different treatment groups. Simultaneously, the heart, liver, spleen, and lungs were fixed in 4% paraformaldehyde solution and embedded in paraffin blocks. Histological observations were subsequently conducted on stained tissue sections.

### Evaluation of anti-inflammatory effects *in vivo*

Blood samples were collected from each group of mice and allowed to stand before being centrifuged at 3000–5000 g for 15 min to isolate the serum. The concentration of the inflammation-related factor IL-6 in the serum was measured using an ELISA kit. Myeloperoxidase (MPO) activity in colon tissues was assessed using a myeloperoxidase kit. Colon tissue samples from different groups were homogenized on ice and centrifuged at 10,000 g for 10 min at 4 °C. The supernatants were collected, and the absorbance at 460 nm was measured using a microplate reader. Malondialdehyde (MDA) activity in colon tissues was analyzed using a malondialdehyde assay kit according to the manufacturer’s protocol.

### Immunohistochemical staining of the colon

Paraffin sections with well-preserved colon morphology were selected for staining to assess the expression of colonic tight junction proteins, including ZO-1 and Occludin. Tissue rehydration is performed after melting the paraffin at a temperature of 65 °C, followed by antigen retrieval. Subsequently, antigen labeling was performed, followed by protein visualization using DAB solution. The expression levels of these tight junction proteins were analyzed using light microscopy across different treatment groups.

### Statistical analysis

Experiments were conducted in triplicate, with results expressed as mean ± standard deviation (SD). Data analysis was performed using Student’s t-test, and significance levels were established at *p < 0.05, **p < 0.01, and ***p < 0.001.

## Results and discussion

### Preparation and characterization of FPPE

**Fig. 2:**
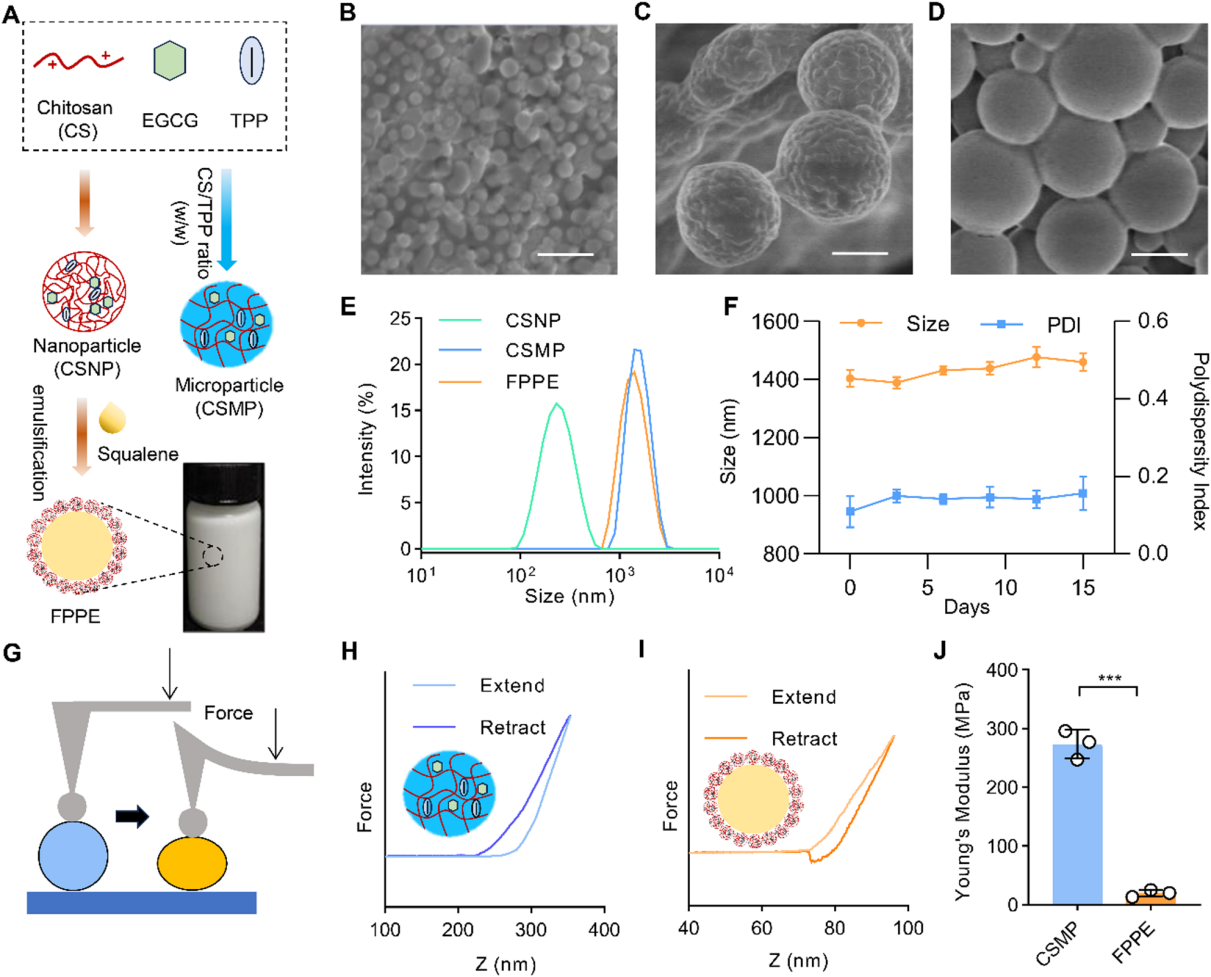
Preparation and characterization of FPPE. (**A**) Schematic illustration of the fabrication process for FPPE and CSMP. (**B**) Scanning electron microscopy (SEM) image of CSNP. Scale bar, 1 μm. (**C**) Cryo-scanning electron microscopy image of FPPE. Scale bar, 1 μm. (**D**) SEM image of CSMP. Scale bar, 1 μm. (**E**) Size distribution of CSNP, CSMP, and FPPE. (**F**) Particle size distribution and polydispersity index (PDI) values of FPPE over 15 days of storage at 4 °C. (**G**) Schematic diagram of the deformation between the AFM tip and the sample. Force-Distance curve measurement experiments of CSMP particles (**H**) and FPPE particles (**I**) as observed via AFM. (**J**) Young’s modulus statistics for FPPE particles and CSMP particles. Data are presented as mean ± SD (*n* = 3). *p < 0.05, **p < 0.01, ***p < 0.001. ns, no significant

The formulation of the FPPE involved a series of reactions, as illustrated in Fig. 2A. First, chitosan nanoparticles (CSNP) loaded with EGCG were formed by ionic cross-linking of chitosan and sodium tripolyphosphate (TPP). The encapsulation efficiency of EGCG was determined to be 89.79 ± 0.08% using high-performance liquid chromatography (HPLC) (Figure S1). Scanning electron microscopy (SEM) confirmed the formation of spherical CSNP, with an average size of 200 nm (Fig. 2B). Subsequently, CSNP were mixed with squalene under ultrasonic conditions to obtain a stable FPPE dispersion. To optimize the particle size and stability of the FPPE, parameters such as particle concentration, ultrasonic power, and ultrasonic duration were systematically adjusted (Figure S2). Cryo-scanning electron microscopy revealed that the FPPE surface exhibited a granule-adsorbed morphology, indicating that FPPE is a particle-stabilized emulsion (Fig. 2C). Meanwhile, chitosan microsphere particles (CSMP) were prepared as a control, and their size and morphology were characterized (Fig. 2D). Particle size distribution analysis indicated that FPPE and CSMP shared similar particle size characteristics (Fig. 2E). In addition, Structured illumination microscopy imaging further confirmed the uniform attachment of CSNP nanoparticles on the surface of FPPE emulsions. (Figure S3). The stability of FPPE was systematically evaluated by monitoring its morphology, particle size, and zeta potential after 15 days of storage at 4 °C and 25 °C (Fig. 2F and Figure S4). The results revealed no noticeable changes in morphology, particle size, or polydispersity index, confirming the high stability of the FPPE formulation under storage conditions. Furthermore, stability testing in simulated gastrointestinal fluids demonstrated that FPPE maintained its structural integrity, further supporting its excellent stability (Figure S5).

In addition, to verify the elastic differences between FPPE and CSMP particles, we conducted a statistical analysis of their elastic moduli using atomic force microscopy (AFM) (Fig. 2G). As shown in Fig. 2H, no evident deflection error was observed, indicating that the CSMP particles remained constant during the process. FPPE particles displayed an evident shift during the retraction process, implying droplet deformation due to probe compression (Fig. 2I). As shown in Fig. 2J, the Hertz model was employed to calculate the elastic properties of the two particles, revealing a significant difference. The Young’s modulus of CSMP microspheres was measured to be 273.33 ± 24.7 MPa, whereas that of FPPE particles was 19.57 ± 5.95 MPa. The substantially higher Young’s modulus of CSMP particles indicates that the material possesses greater rigidity and resistance to deformation. In contrast, the lower Young’s modulus of FPPE particles suggests a softer material with reduced deformation resistance, which is advantageous for applications necessitating higher elasticity or flexibility.

### Mucus penetration ability of FPPE

**Fig. 3:**
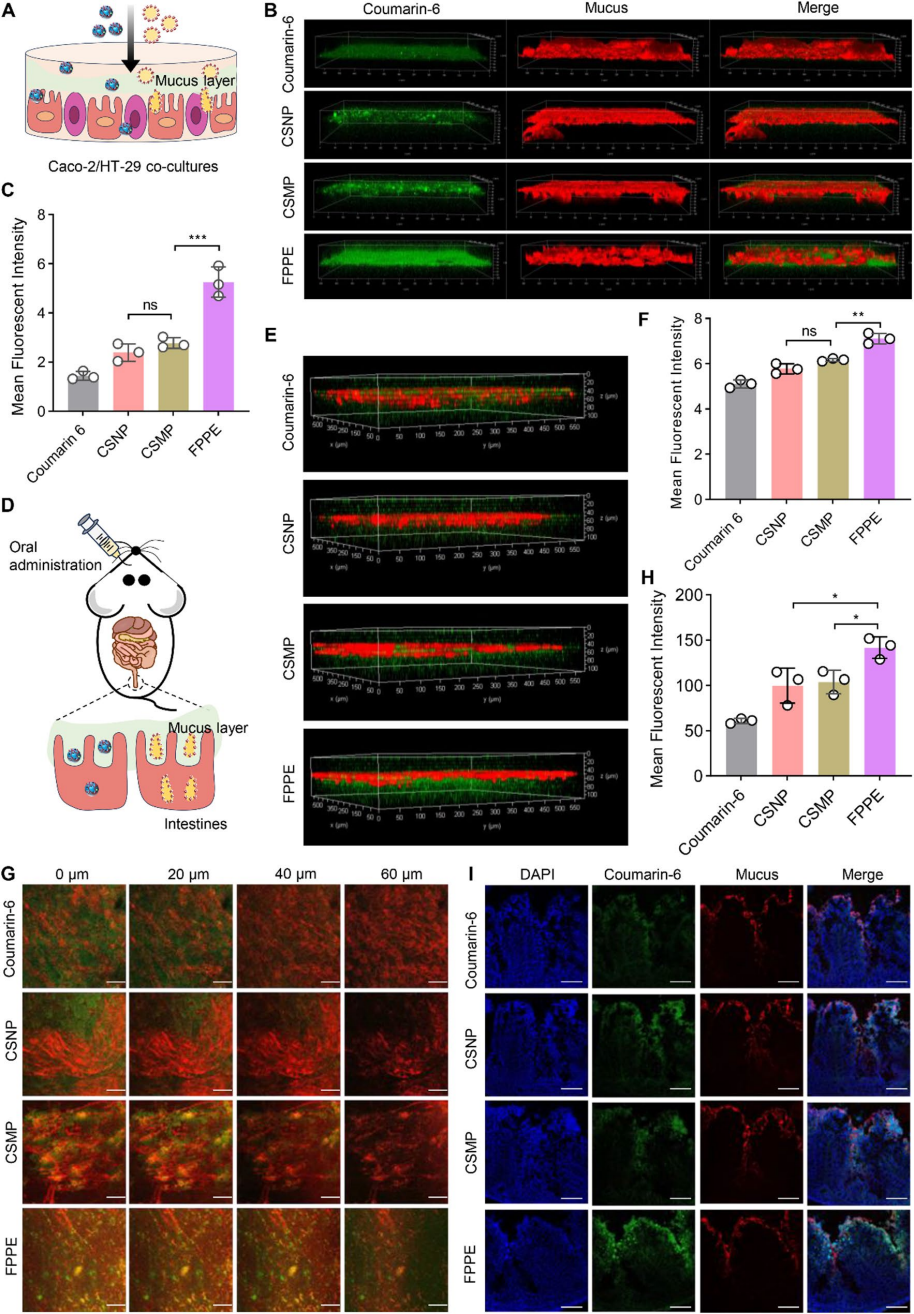
Mucus permeability of FPPE. (**A**) Schematic illustration of Caco-2 and HT-29 cell co-culture model. (**B**) Three-dimensional confocal images of FPPE and CSMP mucus penetration in the co-cultured cell model. Scale bar, 0–100 μm. (**C**) Quantification of penetration retention of FPPE and CSMP in mucus within the co-culture model. (**D**) Schematic representation of particle penetration and uptake of mucus on the surface of intestinal cells in mice. (**E**) Three-dimensional confocal images of FPPE and CSMP mucus penetration in the colon. Scale bar, 0–100 μm. (**F**) Quantification of osmotic retention of FPPE and CSMP particles in intestinal mucus of C57 mice. (**G**) Two-dimensional imaging of FPPE and CSMP infiltration at a depth of 60 μm in the colon. Scale bar, 200 μm. (**H**) Fluorescence quantification of FPPE and CSMP infiltration at a depth of 60 μm in the colon. (**I**) Cryosection analysis of the penetration ability of FPPE and CSMP particles in colon mucus. Scale bar, 200 μm. DAPI, Blue. CSNP-Coumarin 6, CSMP-Coumarin 6, FPPE-Coumarin 6, Green. Mucus stained with Alexa Fluor 647-WGA, Red. Data are presented as mean ± SD (*n* = 3). *p < 0.05, **p < 0.01, ***p < 0.001. ns, no significant

The mucus layer covering the surface of the intestinal epithelium serves as a protective barrier, limiting the penetration of drugs, microorganisms, and toxins [49]. To assess the mucus permeability of FPPE, we used the Caco-2/HT29 co-culture model, which served as an in vitro representation of the intestinal mucus barrier (Fig. 3A). Fluorescence microscopy was used to observe the penetration of coumarin-6-labeled FPPE through the mucus layer in the co-culture system. Both fluorescence imaging (Fig. 3B) and quantitative analysis (Fig. 3C) demonstrated that coumarin-6-labeled FPPE exhibited significantly enhanced mucus permeability compared to coumarin-6-labeled CSMP.

To further investigate mucus penetration by FPPE in vivo, we injected fluorescent-labeled FPPE into the colon of mice and observed fluorescent permeation (Fig. 3D). Three-dimensional imaging demonstrated that FPPE exhibited superior penetration through the mucus layer compared to CSMP (Fig. 3E). Quantitative analysis of the fluorescence intensity within the intestinal mucus further confirmed that FPPE exhibited a higher penetration and retention rate in the colonic mucus than CSMP (Fig. 3F). Additionally, the permeability of fluorescent particles at a depth of 60 μm in the colon was assessed (Fig. 3G-H). The results indicated that the fluorescence intensity of FPPE at 60 μm was also greater than that of CSMP. Collectively, these findings suggest that FPPE possesses enhanced mucus-penetrating capabilities in both the cell co-culture model and the intestinal mucus environment compared to CSMP carriers. This enhanced permeability can be attributed to the rough and flexible surface characteristics of FPPE, which promote efficient adsorption onto the intestinal mucus layer. Additionally, the deformability of FPPE during intestinal peristalsis facilitates its passage through the mucus barrier, further contributing to its superior permeability. Frozen sections of the intestine were prepared to investigate the uptake of FPPE and CSMP by intestinal cells after traversing the mucus layer. As shown in Fig. 3I, the uptake of FPPE by colon cells was significantly greater than that of CSMP, which was consistent with the observed enhanced permeability through the mucus layer.

In summary, the soft and flexible properties of FPPE enabled it to deform under physiological extrusion, facilitating efficient penetration of the mucus barrier. This deformability not only enhances the contact area with intestinal cells, but also improves absorption and uptake efficiency.

### Cell uptake and inflammation inhibition

**Fig. 4:**
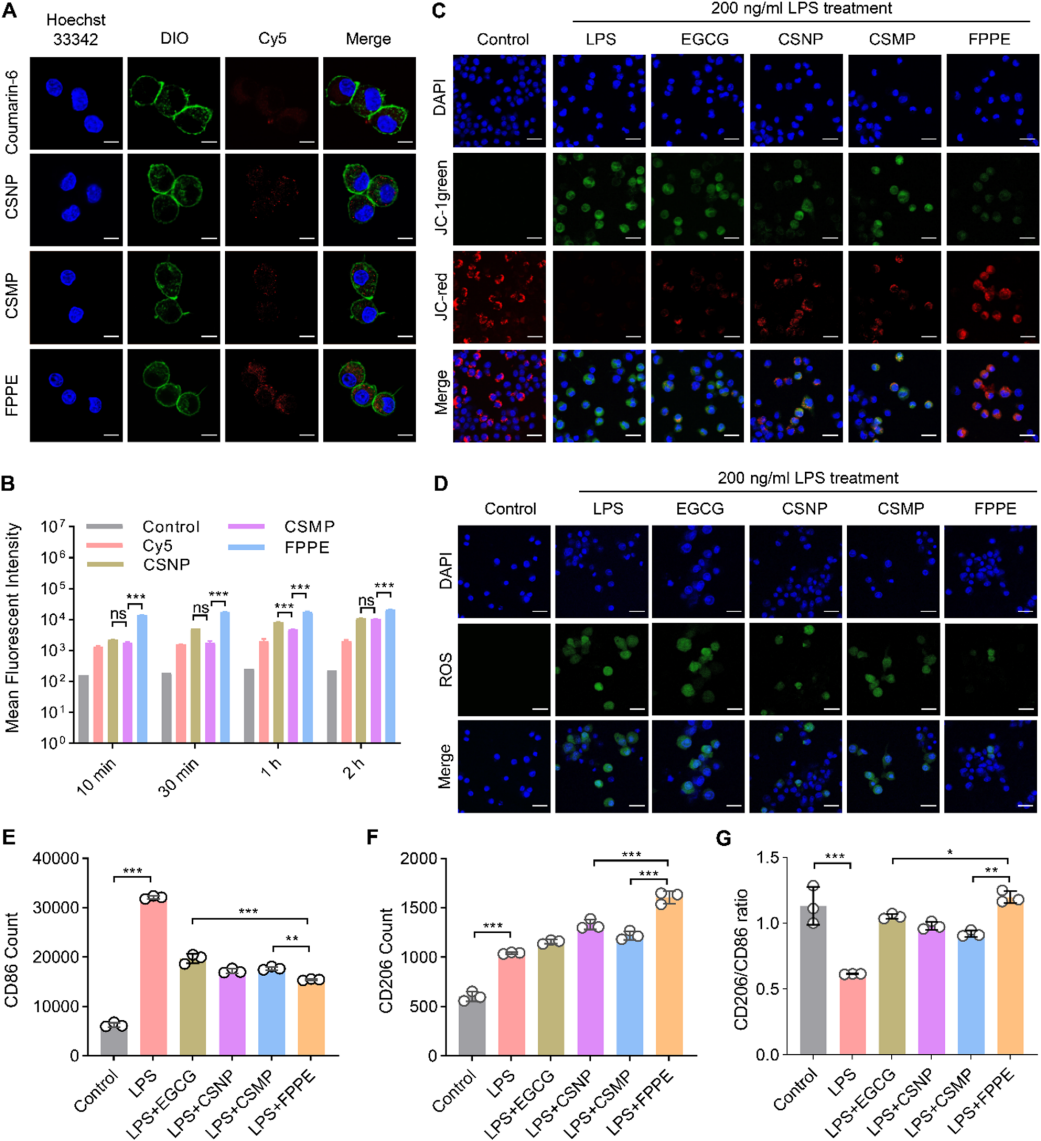
Cellular uptake and inflammation inhibition of FPPE. (**A**) Fluorescence image showing the uptake of FPPE by RAW264.7 cells. Scale bar, 10 μm. (**B**) Flow cytometry analysis of FPPE uptake by RAW264.7 cells at different time periods (10 min, 30 min, 1 h, 2 h). (**C**) Changes in mitochondrial membrane potential following treatment with the indicated formulations. Scale bar, 20 μm. DAPI (blue) indicates cell nuclei. Damaged mitochondria are labeled in green; Healthy mitochondria are labeled in red. (**D**) ROS expression in RAW264.7 cells following treatment with the indicated formulations. Scale bar, 20 μm. DAPI, Blue. ROS, Green. (**E**, **F**) Percentage of M1-type macrophages (**E**) and M2-type macrophages (**F**) determined using flow cytometry. (**G**) Ratio of CD206/CD86. Data are presented as mean ± SD (*n* = 3). *p < 0.05, **p < 0.01, ***p < 0.001. ns, no significant

To investigate the impact of the elastic deformation characteristics of FPPE particles on cellular uptake, RAW264.7 macrophages were cultured to an appropriate density and co-incubated with Cy5-labeled FPPE fluorescent particle carriers for 2 h. The uptake of FPPE by RAW264.7 macrophages was visualized using confocal laser scanning microscopy and quantified using flow cytometry. Fluorescence images revealed that the accumulation of FPPE particles in RAW264.7 cells was significantly greater than that of CSMP particles, indicating a higher uptake of FPPE by macrophages (Fig. 4A). We further quantified the internalization of the FPPE particles at various time points using flow cytometry. As shown in Fig. 4B, the uptake of FPPE particles was significantly higher than that of CSMP particles (*P* < 0.001) after 2 h, confirming the highly efficient cellular uptake of FPPE by RAW264.7 macrophages. Caco-2 intestinal epithelial cells were cultured with coumarin-6-labeled FPPE particles, and particle uptake was quantified using flow cytometry. The results indicated that the FPPE particles were taken up significantly more efficiently than the CSMP particles within 2 h (Figure S6). In conclusion, the flexible extrusion and deformation of FPPE particles increased the contact area between the particles and cells upon mucus penetration, leading to enhanced cellular uptake.

Oxidative stress is a key feature of UC, with reactive oxygen species (ROS) serving as central regulators of inflammatory signaling [50]. Macrophages are essential for maintaining gut homeostasis and for generating immune responses to clear dead cells and pathogens, thereby mitigating inflammation [51]. The anti-inflammatory effects of FPPE were evaluated in vitro using RAW264.7 macrophages. In this study, inflammation was first induced in lipopolysaccharide (LPS)-stimulated RAW264.7 macrophages, followed by co-incubation with FPPE particles for 4 h. Intracellular ROS levels and mitochondrial membrane potentials were assessed using confocal laser microscopy. Mitochondria are major sources of ROS production, and fluorescence images showed that red fluorescence, which is indicative of healthy mitochondrial membranes, was significantly higher in cells treated with FPPE particles than in those treated with CSMP particles, approaching the levels observed in normal controls. Moreover, green fluorescence, which is indicative of compromised mitochondrial membrane potential, was significantly lower in the FPPE group than in the CSMP group (Fig. 4C).

To further assess these anti-inflammatory effects, we evaluated the ability of FPPE and CSMP particles to reduce intracellular ROS. Fluorescence images revealed that green fluorescence, representing ROS expression, was significantly reduced in cells treated with FPPE particles compared with those treated with CSMP particles (Fig. 4D).

In UC, pro-inflammatory M1 macrophages dominate over anti-inflammatory M2 macrophages, leading to impaired intestinal epithelial barrier function and excess pro-inflammatory cytokines [52]. To investigate the effects of FPPE particles on inflammation following cellular uptake by M1 macrophages, we induced an M1 inflammation model in RAW264.7 macrophages using LPS. The macrophages were then co-incubated with FPPE particles to facilitate their polarization toward the M2 anti-inflammatory phenotype. After 4 h, the proportions of M1 and M2 macrophages were assessed using flow cytometry (Fig. 4E-G, Figure S7). The results showed that FPPE particles were more effective than CSMP particles in promoting the conversion of M1 macrophages to the M2 phenotype. In conclusion, our findings demonstrate that FPPE particles efficiently reduce inflammation and oxidative damage in macrophages following cellular uptake.

### Biodistribution of FPPE and CSMP

**Fig. 5:**
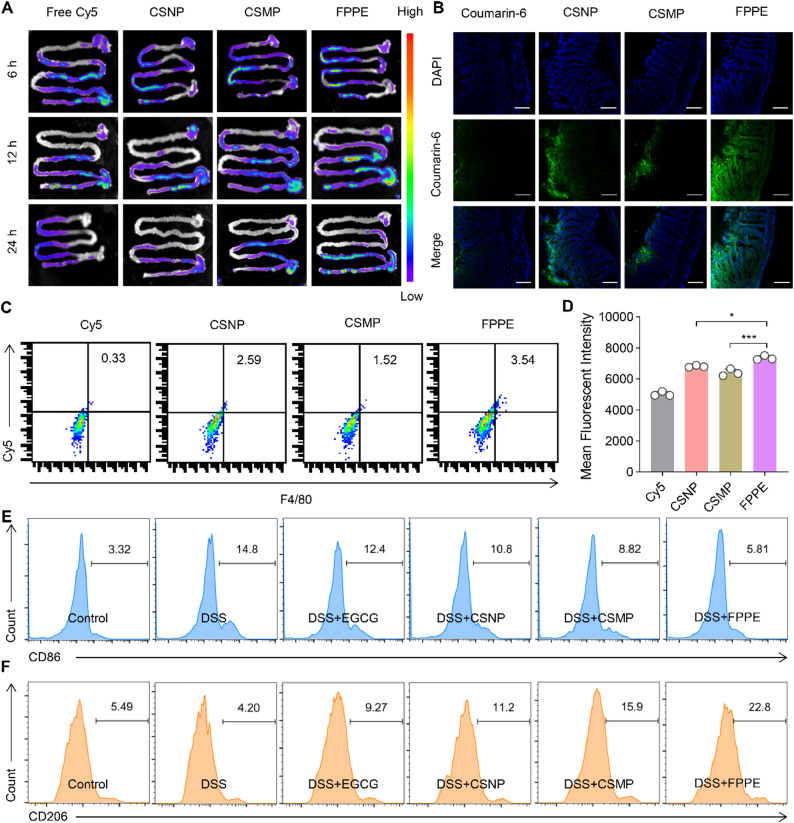
Retention of particles in the gastrointestinal tract of mice. (**A**) Ex vivo fluorescence images of colon and cecum at 6 h, 12 h and 24 h after oral administration of FPPE. (**B**) Absorption of FPPE and CSMP particles in frozen sections of the colon, visualized using laser confocal microscopy. Scale bar, 100 μm. (**C**-**D**) Quantification of macrophage uptake of FPPE and CSMP particles in the colon using flow cytometry. (**E**) Representative flow cytometry data to show the frequency of M1-type macrophages in the colon. (**F**) Representative flow cytometry data to show the frequency of M2-type macrophages in the colon. Data are presented as mean ± SD (*n* = 3). *p < 0.05, **p < 0.01, ***p < 0.001. ns, no significant

Selective accumulation of therapeutic agents in inflamed colonic tissues is crucial for oral drug therapy in UC. After a 12-hour fasting period, Cy5-labeled FPPE particles were orally administered to C57BL6/J mice, with free Cy5 and CSNP solutions as controls. Fluorescence images were acquired to evaluate the retention and distribution of the particles in the gastrointestinal tract (Fig. 5A). Notably, both FPPE and CSMP particles predominantly accumulated in the small intestine 6 h post-administration. After 12 h, both particle types reached the distal small intestine and cecum, with significantly higher fluorescence intensity observed for Cy5-labeled FPPE than for CSMP. After 24 h, the fluorescence from Cy5- and Cy5-labeled CSNP was negligible, whereas Cy5-labeled CSMP showed weak accumulation in the colon. In contrast, Cy5-labeled FPPE particles maintained notable fluorescence intensity in the colon. At the same time, the fluorescence quantification results also showed that the retention of FPPE in the colon after 24 h was higher than that of CSMP (Figure S8).

To further investigate the retention of FPPE particles in the intestine, we sectioned the colon tissue after particle administration and visualized it using laser confocal microscopy. The results revealed that FPPE particles were more efficiently taken up and retained by the colon cells (Fig. 5B). Macrophages isolated from the colon were analyzed for Cy5-labeled FPPE particle uptake using flow cytometry (Fig. 5C-D). Compared with Cy5-labeled CSMP particles, FPPE particles exhibited significantly higher retention in the colon and greater uptake by macrophages. These findings suggest that FPPE particles possess superior mucus permeability, enabling their effective accumulation in the gastrointestinal tract, particularly in the colon, and facilitating enhanced uptake by intestinal cells. In addition, we evaluated the proportion of M1-type (F4/80^+^CD11b^+^CD86^+^) and M2-type (F4/80^+^CD11b^+^CD206^+^) macrophages in the colon via flow cytometry. FPPE treatment significantly decreased the ratio of M1-type macrophages while increasing the ratio of M2-type macrophages, indicating that FPPE facilitated the transition of macrophages from the M1 to the M2 phenotype in the colon tissue (Fig. 5E-F; Figure S9).

### Therapeutic efficacy of FPPE for UC

**Fig. 6:**
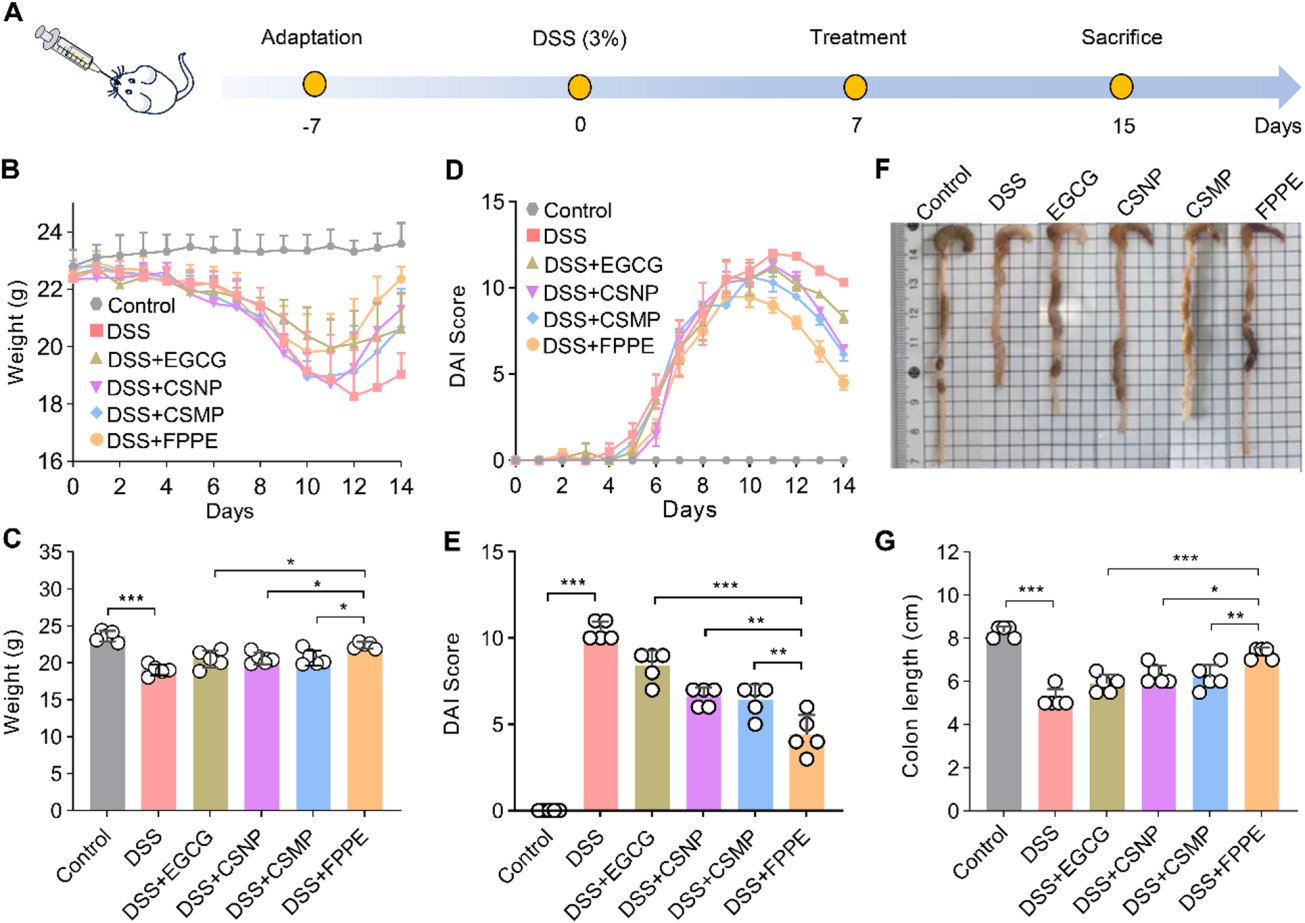
FPPE showed therapeutic efficacy advantages against DSS-induced colitis. (**A**) Overview of DSS induction and drug therapy regimen. (**B**) Changes in daily body weight of mice during induction DSS models and drug treatment. (**C**) Statistics of body weight in each group after the 7th day after intervention. (**D**) Disease activity index (DAI) score during induction DSS models and drug treatment. (**E**) Statistics of DAI score in each group after the 7th day after intervention. (**F**) Representative image of colon length in each group after the 7th day after intervention. (**G**) Statistical analysis of colon length in each group after the 7th day after intervention. Data are presented as mean ± SD. *p < 0.05, **p < 0.01, ***p < 0.001. ns, no significant

We successfully established a murine model of UC using a 3% dextran sulfate sodium (DSS) solution for 7 d, followed by treatment with an FPPE formulation (Fig. 6A). Mice in the UC model exhibited typical pathological features, including hematochezia, body weight loss, and elevated disease activity index (DAI) [53].

Compared to the control group, administration of 3% DSS in drinking water successfully induced colitis, resulting in significant body weight loss compared to the control group. Treatment with EGCG, CSNP, and CSMP partially restored body weight relative to the untreated colitis group. Notably, mice in the FPPE treated group exhibited a more rapid recovery of body weight than those in the CSMP treated group (Fig. 6B-C). In addition, DSS-induced colitis led to severe diarrhea and rectal bleeding, as reflected in the DAI scores. FPPE treatment significantly attenuated diarrhea and rectal bleeding scores, whereas CSMP administration provided only a modest therapeutic effect (Fig. 6D-E). Furthermore, DSS exposure resulted in a marked reduction in colon length, which was most effectively mitigated by FPPE treatment (Fig. 6F-G). Collectively, among the key clinical indicators of UC (body weight loss, diarrhea, rectal bleeding, and colon shortening), FPPE-treated mice demonstrated the most pronounced recovery, suggesting enhanced mucosal healing and attenuation of inflammation.

**Fig. 7:**
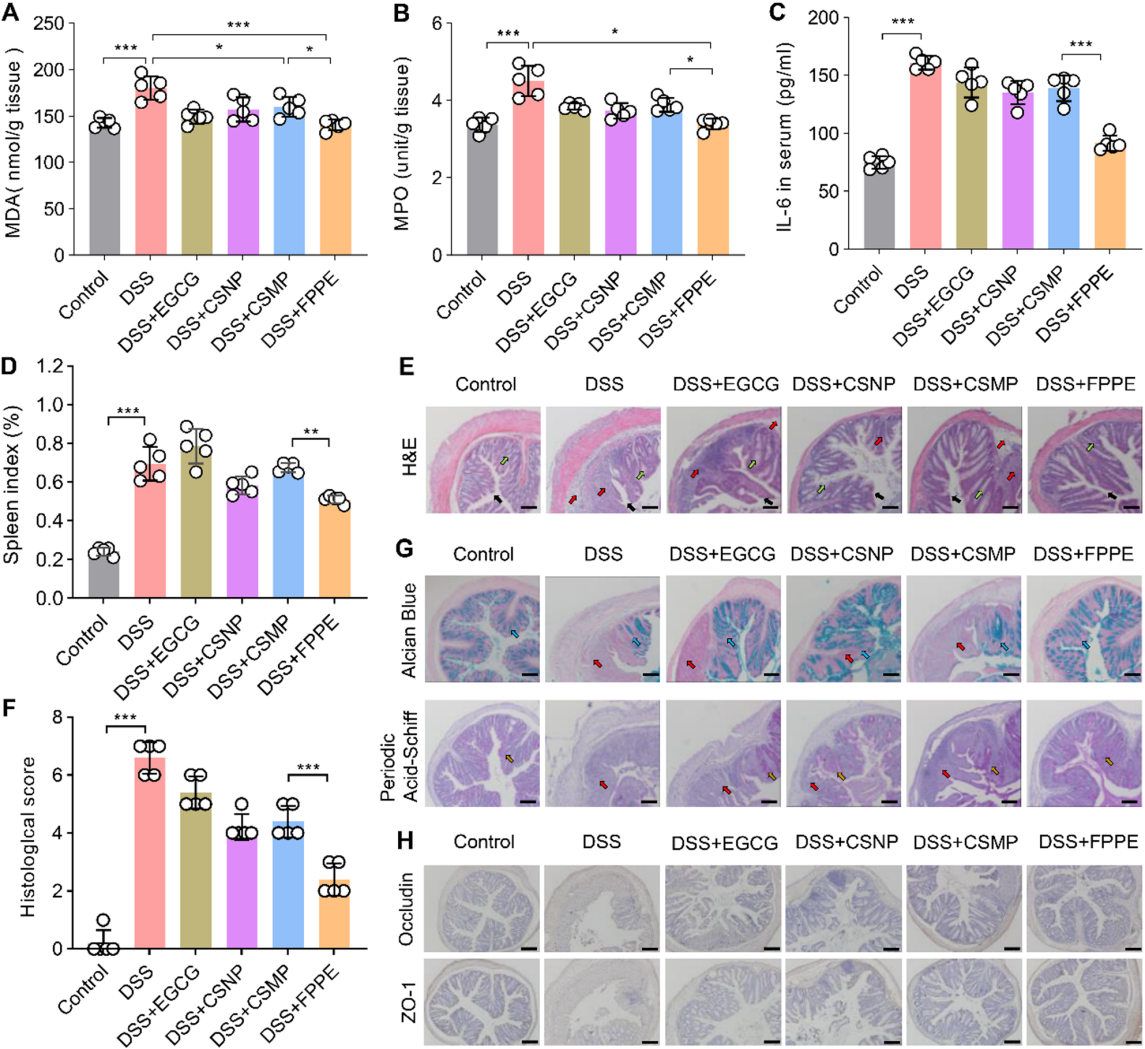
Anti-inflammatory effects and histological evaluation of FPPE in UC. (**A**) Malondialdehyde (MDA) content in colon tissue. (**B**) Myeloperoxidase (MPO) content in colon tissue. (**C**) Serum interleukin-6 (IL-6) levels. (**D**) Statistics of Spleen index after the 7th day after intervention. (**E**) Representative images of H&E staining. Black arrows represent the intestinal epithelial cells in the mucosal layer, red arrows highlight inflammatory infiltrates, green arrows point to the intestinal goblet cells. Scale bar, 50 μm. (**F**) Histological score of colonic H&E staining. (**G**) Alcian Blue staining and Periodic Acid-Schiff staining of different groups. Blue arrows indicate acidic mucin, and yellow arrows mark glycogen. Scale bar, 50 μm. (**H**) Immunohistochemical staining of ZO-1 and occludin proteins in the colon tissue. Scale bar, 50 μm. Data are presented as mean ± SD. *p < 0.05, **p < 0.01, ***p < 0.001. ns, no significant

To further assess the impact of FPPE on colon inflammation, we measured the levels of malondialdehyde (MDA) and myeloperoxidase (MPO) in colon tissues, which are biomarkers of inflammation and neutrophil infiltration in colitis [54]. As shown in Fig. 7A and B, DSS treatment resulted in elevated levels of MDA and MPO, whereas FPPE particle treatment resulted in significantly lower levels of both biomarkers compared to the DSS group. Additionally, serum levels of interleukin-6 were also measured, and the results indicated that FPPE particles effectively inhibited the release of pro-inflammatory cytokines (Fig. 7C).

Compared to the healthy control group, mice in the DSS group exhibited splenomegaly and increased spleen weight due to inflammation, with the most significant reduction in spleen weight observed after treatment with FPPE particles (Fig. 7D). In addition, we assessed the organ indices of the heart, liver, lungs, and kidneys. No significant changes were observed in the heart, lung, or kidney indices (Figure S10). However, liver weight increased owing to the inflammatory response, although this effect was attenuated following FPPE intervention. In addition, histological analysis of major organs (heart, liver, spleen, lung, and kidney) was conducted by hematoxylin and eosin (H&E) staining to assess tissue structural integrity and the extent of inflammatory responses following FPPE intervention (Figure S11). In the DSS-induced colitis model group, the spleen exhibited loosely arranged cells and disrupted architecture, indicating damage to the normal splenic structure and activation of immune responses associated with inflammation. In contrast, all treatment groups showed alleviation of splenic hemorrhage, with the most pronounced improvement observed in the group treated with FPPE emulsion particles, suggesting superior anti-inflammatory efficacy. Additionally, the liver tissues of DSS model mice demonstrated mild swelling, inflammatory cell infiltration, and slight congestion, which were significantly ameliorated after FPPE treatment, reflecting a notable restoration of hepatic health. Histological examination of other organs, including the heart, lungs, and kidneys, revealed normal cellular arrangement and intact tissue architecture, with no evident signs of inflammatory infiltration or pathological alterations, indicating that DSS induction had no significant impact on these organs.

To visually assess the therapeutic effect of FPPE particles on UC, we performed hematoxylin and eosin (H&E) staining of the colon tissue and analyzed the morphological integrity of the colon and the infiltration of inflammatory cells using light microscopy (Fig. 7E-F). Colon tissues from healthy mice exhibited normal histology with intact goblet cells (green arrows), preserved intestinal architecture (black arrows), and no signs of inflammation. In contrast, colon tissues from DSS-treated mice exhibited severe injury, including epithelial cell detachment, intestinal edema, disruption of the crypt structure, a reduction in goblet cell numbers, and substantial immune cell infiltration in the loose connective tissue (red arrows). In the treatment group, damage to the crypt structures was reduced and the number of goblet cells increased. Immune cell infiltration also significantly decreased. Especially, the intestinal mucosa in the FPPE treatment group remained intact, with significantly reduced inflammatory cell infiltration, closely resembling the tissue morphology observed in healthy mice.

In a previous study on UC, AB-PAS staining effectively detected glycogen and polysaccharide components in the colonic mucosa, while distinguishing between neutral and acidic mucins within the same section [55]. This method allows for the assessment of inflammatory response severity and mucosal repair status. In this study, alcian blue (AB) staining (pH = 2.5) highlighted acidic mucilage substances (Fig. 7G). Compared with the healthy control group, DSS treatment significantly damaged the mucus barrier, leading to reduced mucus content. In contrast, mice treated with FPPE particles showed gradual recovery of the intestinal mucus barrier, with the mucus content approaching that of healthy mice. Periodic acid–Schiff (PAS) staining revealed that FPPE particle-treated mice had a normal proportion of regenerated colonic crypts, intact mucus-producing goblet cell morphology, and glycogen content comparable to that of healthy mice. These findings suggest that FPPE carriers have a greater potential than CSMP particles for repairing colonic injuries.

Immunohistochemical staining of tight junction proteins demonstrated a marked disruption of intestinal barrier integrity in the DSS group, where ZO-1 and occludin were loosely arranged or even absent [56]. In contrast, FPPE treatment effectively preserved the barrier structure, with the distribution of tight junction proteins in the colon closely resembling that of healthy controls (Fig. 7H). Consistently, immunofluorescence analysis further confirmed that FPPE administration markedly restored the expression and localization of ZO-1 and occludin, indicating its strong protective effect on the intestinal epithelial barrier (Figure S12).

In conclusion, the histochemical staining results demonstrated that FPPE flexible carriers effectively reduced pro-inflammatory factor production in UC. In addition, FPPE particles promoted efficient penetration and accumulation at the site of intestinal injury, restored the physiological function of tight junction proteins, and repaired the intestinal mucosal barrier.

## Conclusion

This study highlights the importance of flexible contact properties in oral drug delivery systems and demonstrates the superiority of FPPE carriers for intestinal barrier penetration and drug accumulation. Through comparative analysis with rigid CSMP, we observed that the deformation ability of FPPE enabled more effective penetration of the intestinal mucus layer and enhanced accumulation at sites of injury. This advantage is attributed to the high contact area and physiological squeezing effect of FPPE, which improves targeting and bioavailability compared to rigid carriers. Our results clearly demonstrate that FPPE carriers exhibit notable mucus penetration and cellular uptake, making them promising candidates for treating intestinal diseases. Moreover, this study provides a conceptual framework for the design of oral delivery carriers, suggesting that flexible carriers may offer significant advantages, particularly in treating gastrointestinal diseases.

Although FPPE carriers exhibit clear advantages in mucus penetration, the underlying mechanisms remain unclear. Further studies are needed to explore how these carriers interact with mucins in the intestinal mucosal layer and how these interactions affect drug release and absorption. Specifically, the deformation and adhesive properties of flexible carriers may influence penetration rates and bioavailability, highlighting the importance of understanding these interactions at the molecular level to optimize drug delivery systems. Our study confirmed the high efficacy of FPPE carriers in penetrating the intestinal mucus layer. However, further research is needed to evaluate carriers with varying rigidity and elasticity. Comparative studies on soft, medium-hard, and rigid carriers could provide insights into their optimal design for oral drug delivery, with soft carriers suitable for superficial layers, rigid carriers suitable for thicker mucus layers, and medium-hard carriers offering a balance between penetration and stability [57]. One potential approach is to examine carriers with intermediate rigidity by systematically modulating nanoparticle shell density or the oil-to-nanoparticle ratio in the Pickering emulsions. The mechanical characteristics of these carriers could be quantified using atomic force microscopy (AFM) or micropipette aspiration, which directly assess deformability under physiologically relevant forces. Their subsequent penetration capacity can then be evaluated in in vitro mucus models or ex vivo tissue assays. Correlating deformability with penetration efficiency would provide a more precise understanding of the flexibility threshold required for effective mucus transport. In addition, in drug delivery system design, factors such as particle size, surface charge, shape, and deformability critically affect drug release and biocompatibility. The deformation properties of FPPE carriers may yield varying effects under different physiological conditions, necessitating research on their performance under pathological conditions, such as inflammation or tissue injury. Such investigations are vital to optimize drug delivery systems and improve the treatment of gastrointestinal diseases.

During the scale-up production of FPPE, ensuring uniform decoration of nanoparticles on the emulsion surface is a critical challenge, as it directly affects both the stability and functionality of the emulsions. At the same time, precise control of droplet size distribution is essential, since heterogeneity in droplet size can reduce drug-loading efficiency and promote sedimentation. The physicochemical stability of FPPE during storage and handling is also crucial, including preventing droplet aggregation, phase separation, and maintaining the integrity of nanoparticle decoration. In addition, multiple process parameters, such as mixing speed, emulsification energy, and temperature, require careful optimization, as they not only influence the initial emulsion structure but also affect long-term stability and reproducibility. For scale-up production, establishing standardized operating procedures combined with process optimization strategies is necessary to ensure that high drug-loading efficiency, uniform droplet size, and stable nanoparticle decoration are maintained under large-scale conditions.

## Supplementary Information

Supplementary Material 1

## Data Availability

Data is provided within the manuscript or supplementary information files.
