# Supplementary material for: Flexibility-powered Pickering emulsion enhances mucus permeability to alleviate ulcerative colitis
Source: J Nanobiotechnology. 2025 Dec 23;23:784. doi: 10.1186/s12951-025-03829-6 (PMC12729323; doi:10.1186/s12951-025-03829-6)
Supplement: Supplementary file 1 — Supplementary Material 1 [file 12951_2025_3829_MOESM1_ESM.pdf]

Supplementary Information

Flexibility-powered Pickering emulsion enhances mucus permeability to alleviate ulcerative colitis

Authors

Jiali Lv[1†], Chen Cheng[2†], Xinran Liu[1], Jinhua Song[1], Daxiang Li[1], Yijun Wang[1], Yiqun Du[1]*

Affiliations

[1] State Key Laboratory of Tea Plant Germplasm Innovation and Resource Utilization, School of Food and Nutrition, Anhui Agricultural University, Hefei 230036, China

[2] Institute of Clinical Immunology, the First Affiliated Hospital of Anhui Medical University, Anhui Medical University, Hefei 230032, China

[†] Jiali Lv and Chen Cheng have contributed equally to this work.

*Corresponding author.

E-mail: Yiqun Du, duyiqun@ahau.edu.cn.

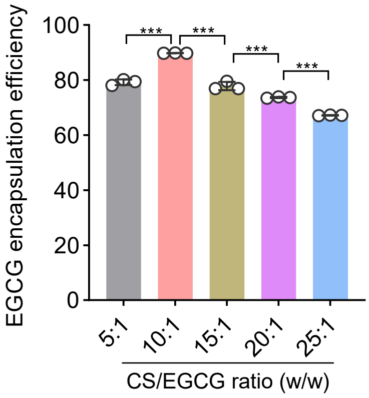

Figure S1. Encapsulation efficiency of EGCG in CSNP particles. The CS/EGCG ratio of 10:1 (w/w) was selected for further experiments.

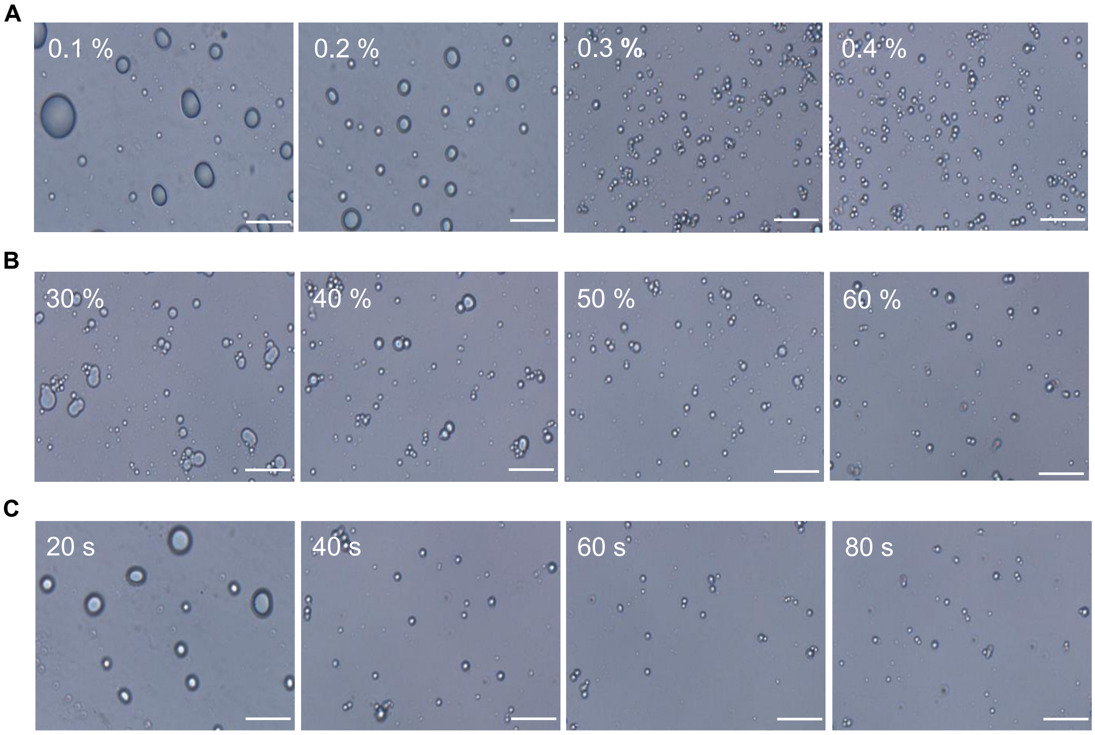

Figure S2. Optical morphology images of FPPE droplets prepared under different CS concentrations (A), ultrasonic amplitudes (B) and ultrasonic times (C). Scale bar, 10 μm. The 0.3 % mass concentration, 60 % ultrasonic amplitude, and 60 s ultrasonic time were selected for subsequent experiments.

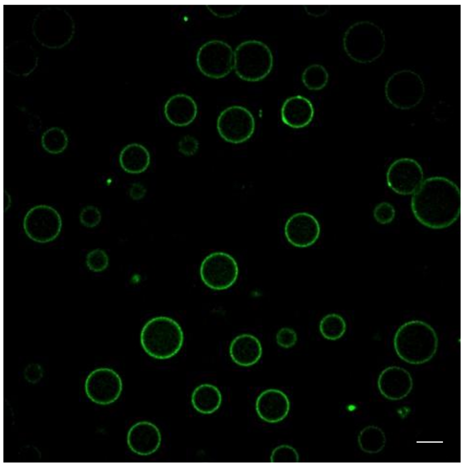

Figure S3. Structured illumination microscopy image of FPPE emulsion droplets. Scale bar, 1 μm.

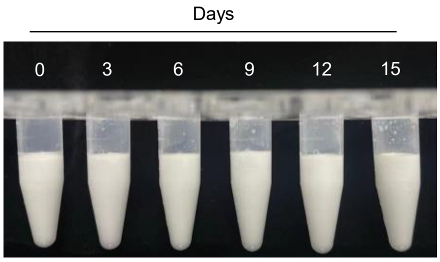

Figure S4. Morphological changes of nanoparticles after 15 days of storage at 25°C.

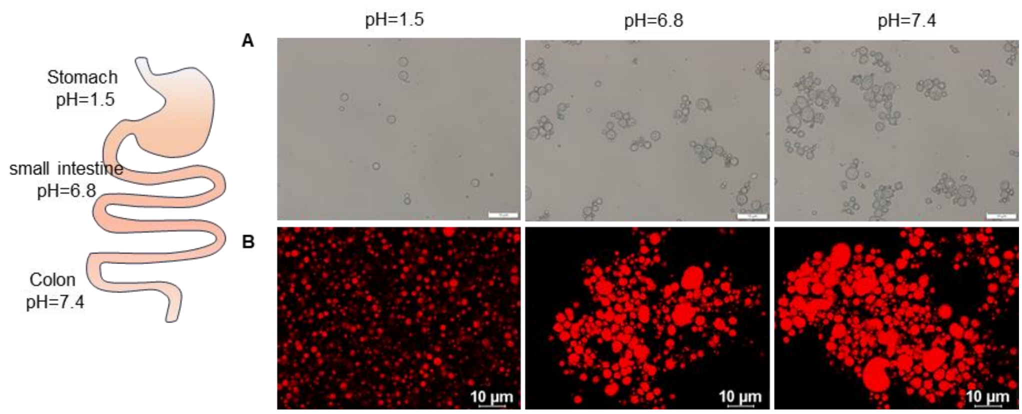

Figure S5. Optical morphology of FPPE emulsions in simulated gastric, intestinal, and colonic fluids. (A) Optical microscopy images. (B) Confocal laser scanning microscopy images.

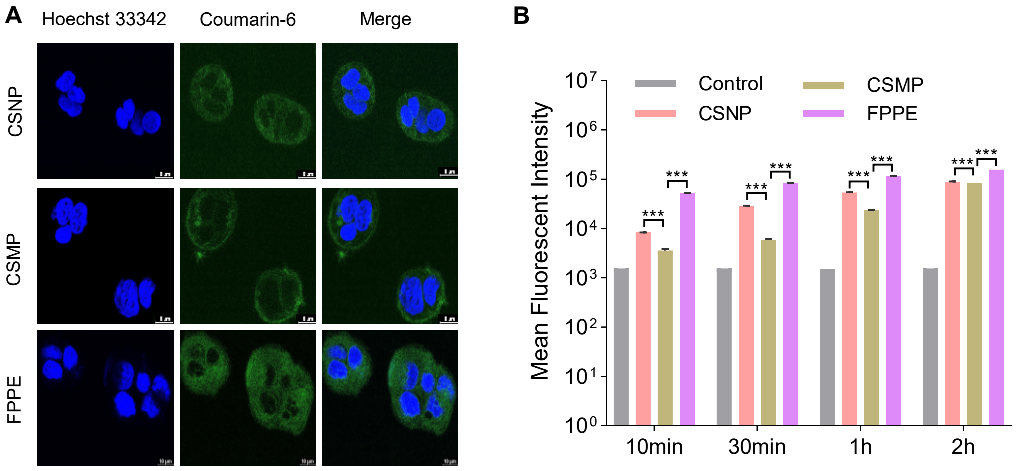

Figure S6. (A) Fluorescence image of Caco-2 cells absorption. Scale bar, 10 μm. (B) Flow cytometry analysis of Caco-2 cell uptake at various time points (10 min, 30 min, 1 h, and 2 h).

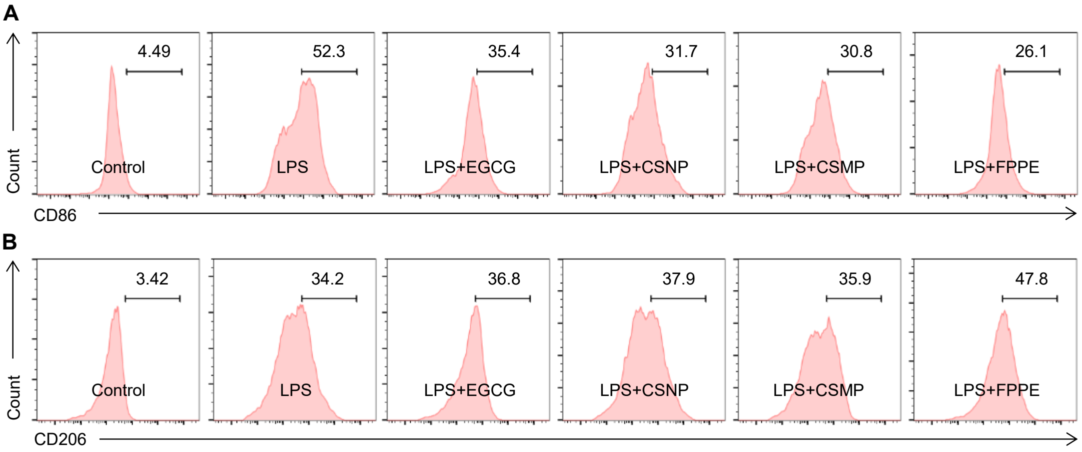

Figure S7. Percentage of CD86^{+} cells and CD206^{+} cells.

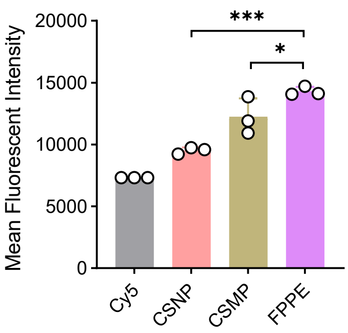

Figure S8. The distribution of FPPE and CSMP particles in the intestinal tract was quantified by fluorescence at 24 hours after oral administration.

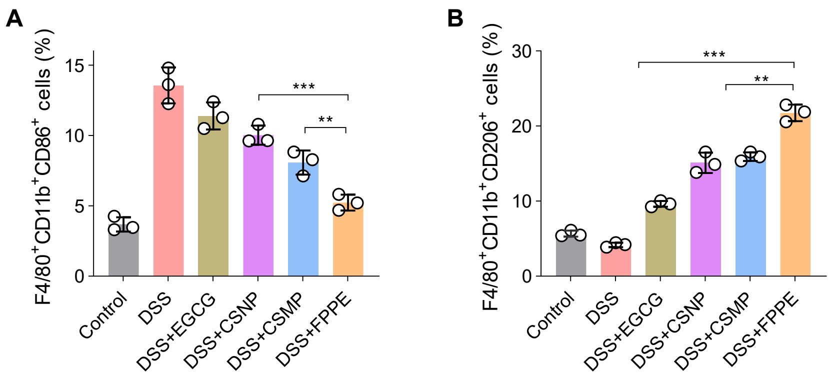

Figure S9. Percentages of M1-type macrophages (F4/80$^+$CD11b$^+$CD86$^+$ cells) and M2-type macrophages (F4/80$^+$CD11b$^+$CD206$^+$ cells) in the colon determined by flow cytometry.

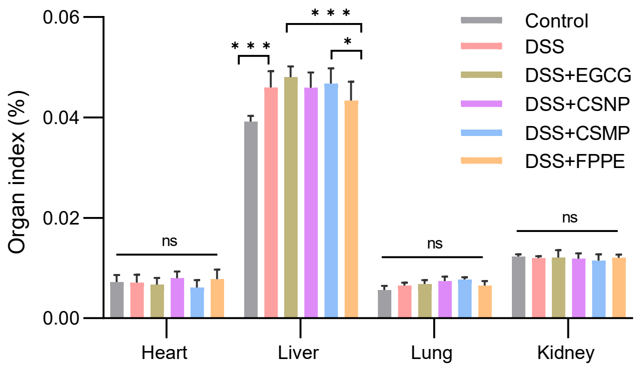

Figure S10. Evaluation of the therapeutic efficacy of FPPE intervention for ulcerative colitis based on heart, liver, lung, and kidney indices.

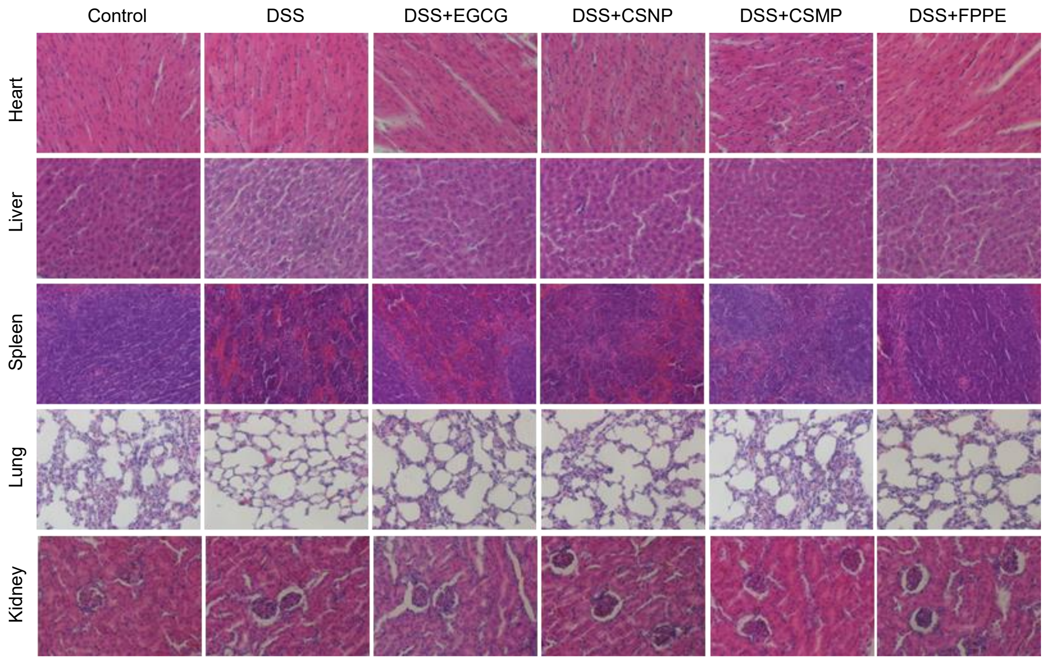

Figure S11. Hematoxylin eosin staining of heart, liver, spleen, lung and kidney of colitis mice after FPPE intervention.

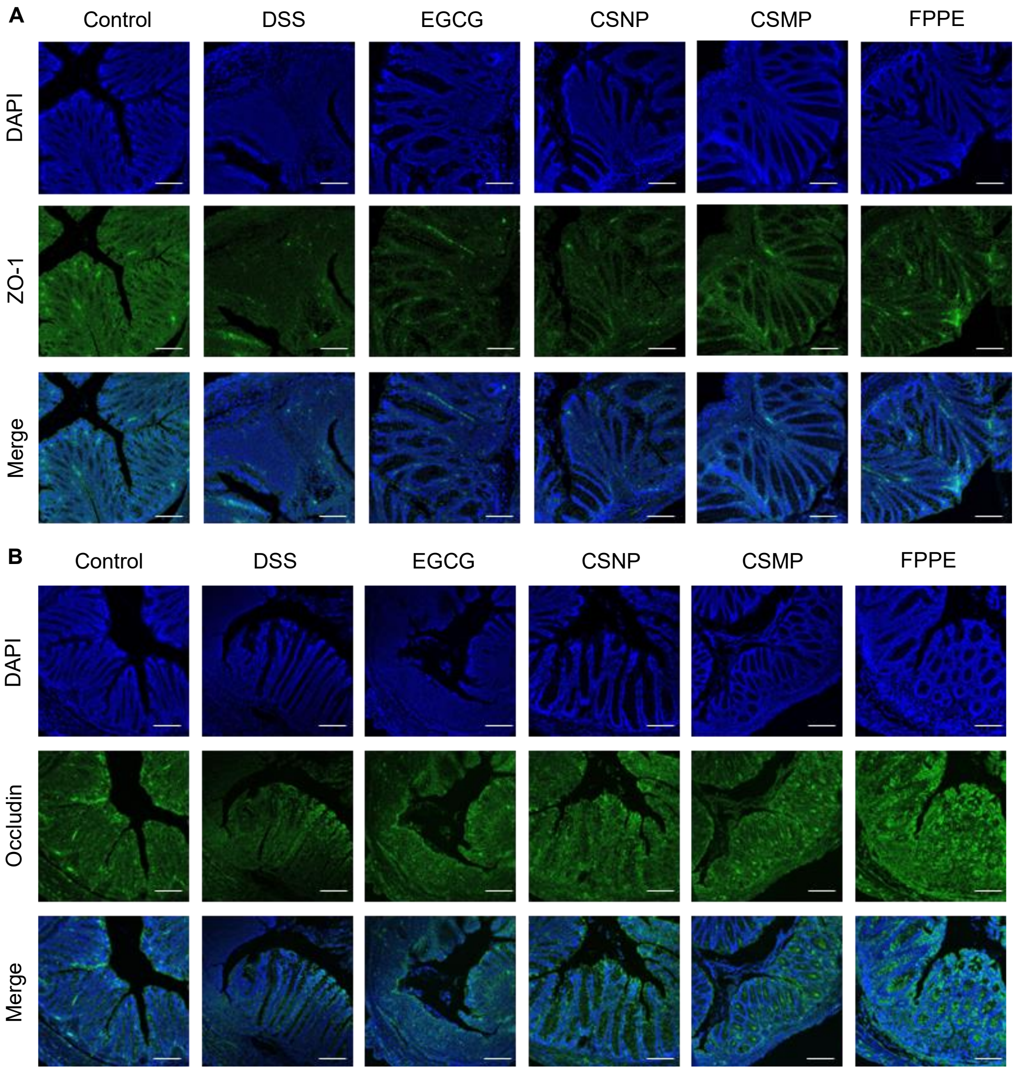

Figure S12. Immunofluorescence analysis of ZO-1 (A) and Occludin (B) protein distribution in colon tissue. Representative images show the localization of ZO-1 and Occludin in colon sections. Nuclei were stained with DAPI (blue). Scale bar, 100 μm.
